# Network-level analysis of metabolic regulation in the human red blood cell using random sampling and singular value decomposition

**DOI:** 10.1186/1471-2105-7-132

**Published:** 2006-03-13

**Authors:** Christian L Barrett, Nathan D Price, Bernhard O Palsson

**Affiliations:** 1 Bioengineering Department, University of California – San Diego, 9500 Gilman Drive, La Jolla, California, 92093-0412, USA; 2 Institute for Systems Biology, 1441 North 34th Street, Seattle, WA, 98103, USA

## Abstract

**Background:**

Extreme pathways (ExPas) have been shown to be valuable for studying the functions and capabilities of metabolic networks through characterization of the null space of the stoichiometric matrix (**S**). Singular value decomposition (SVD) of the ExPa matrix **P **has previously been used to characterize the metabolic regulatory problem in the human red blood cell (hRBC) from a network perspective. The calculation of ExPas is NP-hard, and for genome-scale networks the computation of ExPas has proven to be infeasible. Therefore an alternative approach is needed to reveal regulatory properties of steady state solution spaces of genome-scale stoichiometric matrices.

**Results:**

We show that the SVD of a matrix (**W**) formed of random samples from the steady-state solution space of the hRBC metabolic network gives similar insights into the regulatory properties of the network as was obtained with SVD of **P**. This new approach has two main advantages. First, it works with a direct representation of the shape of the metabolic solution space without the confounding factor of a non-uniform distribution of the extreme pathways and second, the SVD procedure can be applied to a very large number of samples, such as will be produced from genome-scale networks.

**Conclusion:**

These results show that we are now in a position to study the network aspects of the regulatory problem in genome-scale metabolic networks through the use of random sampling.

**Contact**: palsson@ucsd.edu

## Background

In recent years, chemically-detailed metabolic networks capable of simulating growth have been reconstructed at the genome-scale for nearly twenty organisms [1]. Any metabolic network can be represented as a stoichiometric matrix, and by assuming steady-state conditions one can compute the "space" in which all allowable steady-state flux distributions reside. This space can be spanned by a set of extreme pathways (ExPas) [2,3], and for a given metabolic network this set is unique, invariant and irreducible [4]. ExPas are so named because they form the edges of the steady-state solution space of the network and thus characterize its "extreme" functions. Mathematically, the ExPas are a set of conical basis vectors that forms a convex cone in high-dimensional space. In this manner, the extreme pathways circumscribe all of the possible steady-state flux distributions possible in the metabolic network. Biochemically, each ExPa corresponds to a steady-state flux distribution through the network, and is more than just a linear set of reactions linking substrate to product. Specifically, each ExPa describes the relative fluxes among a set of reactions needed for the conversion of substrate(s) into product(s), and does so by creating all byproducts needed to maintain systemic balances and by simultaneously maintaining all cofactor pools at steady state. The development of such network-based pathway definitions, including the elementary flux modes [5-7], has led to a systems view of network properties [8]. Genome-scale metabolic pathway analysis [5,9,10] holds much promise for understanding fundamental aspects of network functionality, robustness, and regulation [11,12], for metabolic engineering [13,14], and for elucidating many other systems properties [12,15-23].

The number of ExPas for a reaction network grows exponentially with network size, limiting their use for the analysis of large networks. Because of this limitation, ExPas have mainly been used to analyze realistic, but small, networks such as the hRBC [22]. In further work [4,24], singular value decomposition (SVD) was applied to the ExPa matrices (**P**), resulting in two key insights. First, it was shown that SVD of **P **can compute the effective dimensionality of the regulatory problem from a network perspective. Second, it was shown that the 'eigenpathways' that correspond to the modes of the SVD identified key network branch points that can represent key control points (reactions) in the network. These points constitute a minimal set of reactions whose control sets the steady state flux state for the entire network, represented as a specific location in the steady-state solution space. SVD rank orders these key branch points in accordance with the shape of the solution space and thus rank orders the effectiveness by which the branch points can be used to move the network's flux distribution around in the solution space. These features were demonstrated by showing that the dominant features of the first five eigenpathways correspond to actual major points of control in the hRBC and can effectively locate the solution in a 5-dimensional space [24]. SVD of **P **can thus define the magnitude and nature of the regulatory problem associated with controlling a metabolic network.

Given the fundamental understanding of network properties that can be derived from the SVD of **P**, it would be natural to perform the same analysis on the much larger genome-scale metabolic networks that have already been reconstructed. Unfortunately, this is not currently possible due to the NP-hard nature of calculating ExPas. Even if computing ExPas were possible on a genome-scale, the likely result would be matrices containing extremely large numbers of ExPas, many more than would be needed using uniform random sampling. This number of ExPas presents an additional formidable hurdle for the SVD calculation, which is an O(n^2^) algorithm [25].

In this work, we demonstrate an approach that addresses this problem. As mentioned above, the set of ExPas for a metabolic network defines the shape of the steady-state solution space of the network. The information that SVD of the set of ExPas provides is a description of the space. The eigenvectors that are produced by the SVD indicate, in rank order, the directions corresponding to the greatest variance in the high-dimensional space, and the singular values give the relative magnitude of this variation. A potentially better approach is to characterize the shape of the solution spaces by generating uniform random samples within it. Each sample solution is used as a column in a matrix (**W**). With enough samples, a "picture" of the solution space emerges, which can then be analyzed using SVD in the same manner as was done previously for the ExPas. In this work, we performed uniform random sampling of the steady-state solution space of the hRBC metabolic network and applied SVD to the matrix, **W**, of these samples. We show that similar, though not identical, results are obtained through SVD of **W **as were obtained through SVD of **P**. Thus, this study provides a new method for the calculation of Principle Regulatory Modes (PRMs) that improves upon the previous method by being more tractable for genome-scale networks due to its independence from ExPas. Additionally, the interpretation of the results of the new method are more straightforward because it characterizes the shape of the solution space uniformly, without being affected by the non-uniformity of the distribution of ExPas on the edges of the solution space.

## Results

### Ascertaining sampling sufficiency

**Figure 1:**
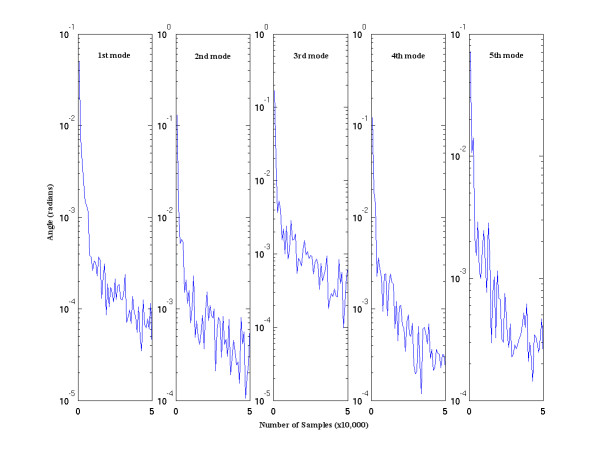
The SVD procedure was applied for increasingly larger sample sizes (up to 1,000,000 in increments of 2,000) and the first five modes at each sample size were recorded. These plots show the convergence of the angle between the modes for subsequent sample sizes.

We first determined that the steady-state solution space of the hRBC was thoroughly sampled and that an accurate set of solutions had been sampled. Incomplete sampling would have the effect of misrepresenting the shape of the space and would lead to inaccurate results. We determined the thoroughness of the sampling by plotting the angle (see Methods section) between subsequent SVD modes for increasing sample sizes. This convergence is shown in Figure 1 for the five modes with the largest singular values. As can be seen, convergence occurred after using just a few thousand sample solutions, with very little change over the remainder of the one million samples. This result demonstrates that the solution space of a small metabolic network, which in the hRBC contains just 51 reactions, can be studied with relatively few samples.

### Comparing the modes derived from P and W

**Figure 2:**
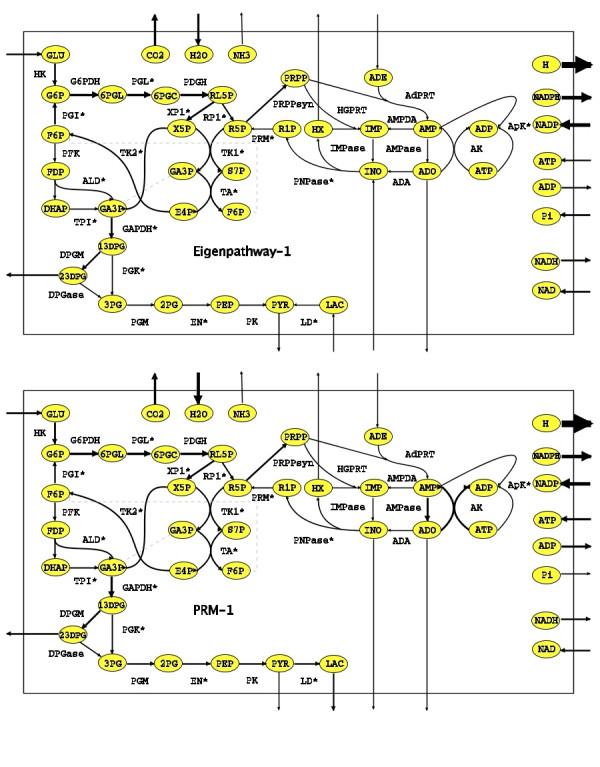
The first eigenpathway derived from SVD of the extreme pathways matrix and the first PRM derived from SVD of the samples matrix for the hRBC metabolic network. The relative values of the flux values is indicated by the thickness of the arrows between metabolites. The metabolite and reaction definitions are given in Table 1 and Table 2.

**Figure 3:**
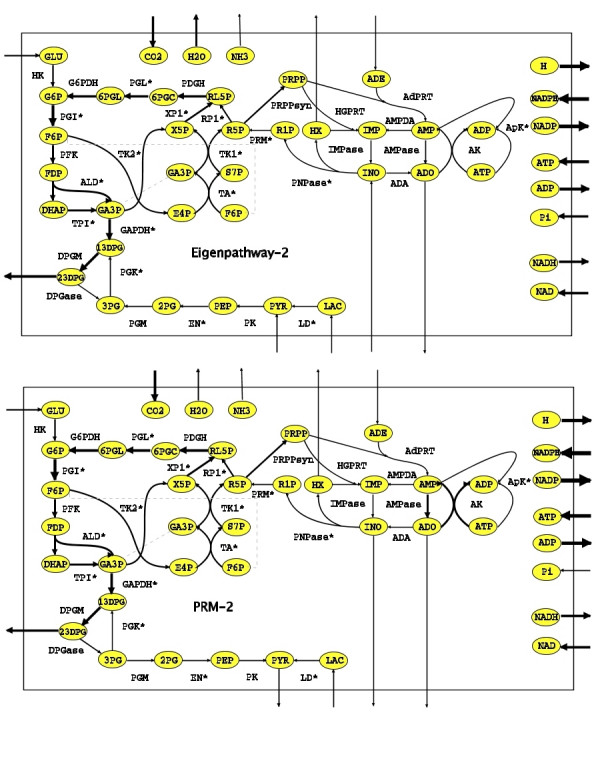
The second eigenpathway derived from SVD of the extreme pathways matrix and the second PRM derived from SVD of the samples matrix for the hRBC metabolic network. The relative values of the flux values is indicated by the thickness of the arrows between metabolites. The metabolite and reaction definitions are given in Table 1 and Table 2.

**Figure 4:**
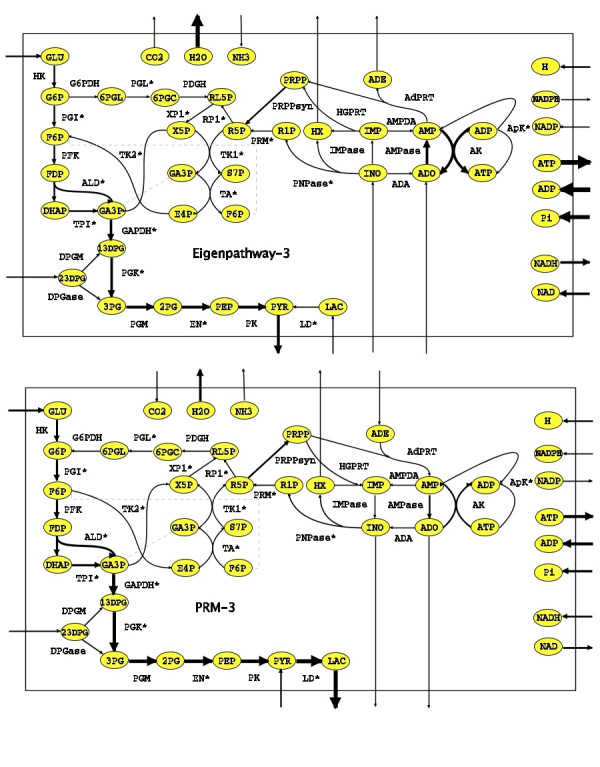
The third eigenpathway derived from SVD of the extreme pathways matrix and the third PRM derived from SVD of the samples matrix for the hRBC metabolic network. The relative values of the flux values is indicated by the thickness of the arrows between metabolites. The metabolite and reaction definitions are given in Table 1 and Table 2.

**Figure 5:**
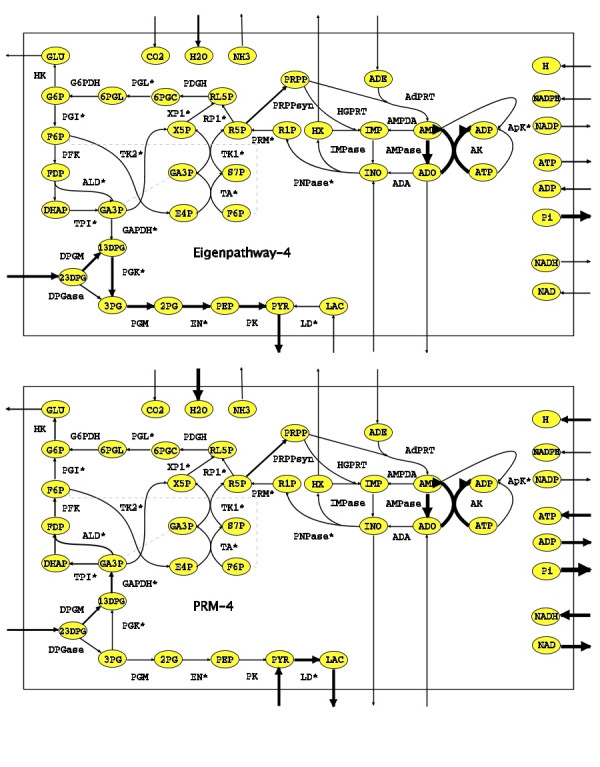
The fourth eigenpathway derived from SVD of the extreme pathways matrix and the fourth PRM derived from SVD of the samples matrix for the hRBC metabolic network. The relative values of the flux values is indicated by the thickness of the arrows between metabolites. The metabolite and reaction definitions are given in Table 1 and Table 2.

**Figure 6:**
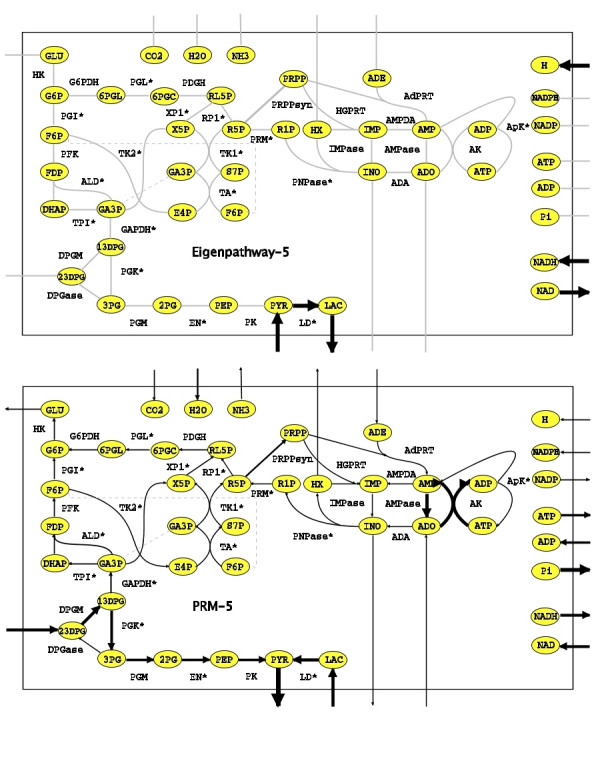
The fifth eigenpathway derived from SVD of the extreme pathways matrix and the fifth PRM derived from SVD of the samples matrix for the hRBC metabolic network. The relative values of the flux values is indicated by the thickness of the arrows between metabolites. The metabolite and reaction definitions are given in Table 1 and Table 2.

The modes corresponding to the first five singular values obtained from SVD of the extreme pathways (eigenpathways) and from the samples (principal regulatory modes, or PRMs) were shown on a flux map of the hRBC. These maps were then compared by placing them side-by-side in Figures 2, 3, 4, 5, 6. The differences and similarities between the eigenpathways and PRMs are addressed in the Discussion section.

### Assessing the correspondence of inferred reaction regulatory importance

One of the primary insights resulting from the application of SVD to **P **was an understanding of the relative importance amongst all of the reactions in terms of regulatory control. The aim of this work was to show that similar insight can be obtained through uniform random sampling. To do this, we followed a previously developed procedure [26] and scored each reaction by the *l*^2^-norm (length) of its loading vector, which is a vector of the reaction's contribution to each of the eigenvectors resulting from the SVD procedure (see Methods section). The rationale for this scoring method is based on our goal of identifying those reactions most critical for controlling flux through the metabolic network. These control reactions are expected to have larger contributions to the eigenvectors resulting from the SVD procedure. The impact of these larger contributions is reflected in a larger absolute value of the loading vector, signifying a larger distance of the reaction from the origin. So in this context the *l*^2^-norm quantifying the length of the loading vector is appropriate for our purposes.

Scores allow us to impart a rank ordering on the reactions. The degree to which the SVD of **W **and the SVD of **P **provide similar information about the regulatory importance of the reactions, then, is measured by the similarity in which they rank order the reactions by their scores. We measured this similarity by computing the rank correlation coefficient between the two rank orderings of reactions. Just like Pearson's correlation coefficient, the rank correlation has a range [-1, 1]. We used two related rank correlation measures [27]: Kendall's tau and Spearman's rho. We found the reaction rankings to be highly correlated, resulting in a tau value of 0.88 with P-value 1.1*10^-15 ^and a rho value of 0.97 with P-value 4.0*10^-12^. Based on these results, we claim that the sampling methodology produces similar insight into identifying the most important reactions for flux control as did the ExPa-based approach.

### Reconstruction of physiological flux distributions

**Figure 7:**
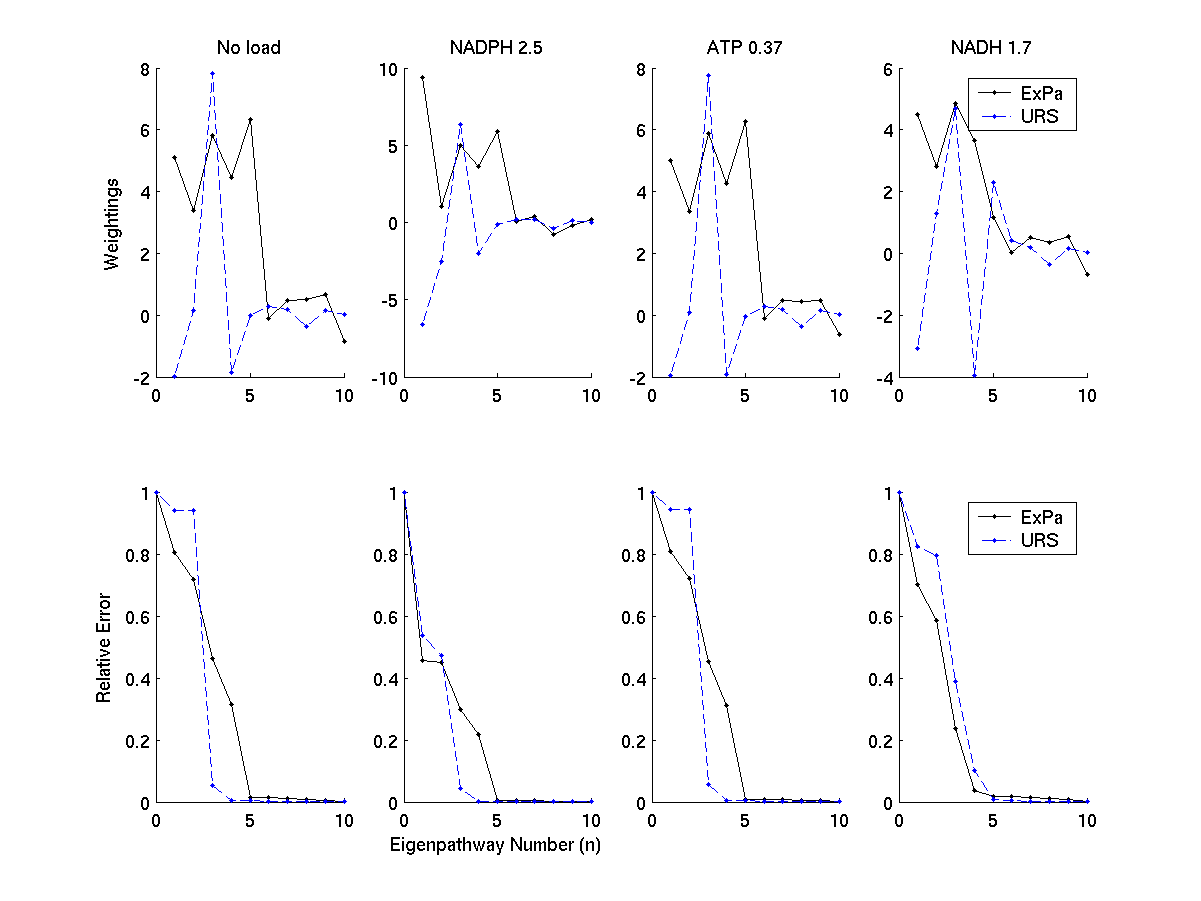
A comparison of how the eignpathways and PRMs reconstruct four different physiological flux distributions in the hRBC. The upper plots show the mode weightings for reconstructing the flux distributions, and the lower plots show the relative error between the reconstructed and physiological flux distributions for increasing numbers of modes.

We determined how the modes computed from SVD of **W **were combined to reconstruct the physiological flux distributions and how the difference between the reconstructed and actual distributions diminished as more singular modes were used to reconstruct the desired solution. The reconstruction and difference computations were performed in the same manner as described previously (Price, Reed et al. 2003). Additionally, we assessed how the efficiency of these reconstructions compared with those based on the eigenpathways. The results are shown in Figure 7. These results indicate that in 3 out of the 4 cases, fewer of the PRMs are required for reconstructing the flux distributions than were required using eigenpathways. In the fourth case, the same number of modes yields similar reconstruction accuracy.

### Genome-scale sampling

**Figure 8:**
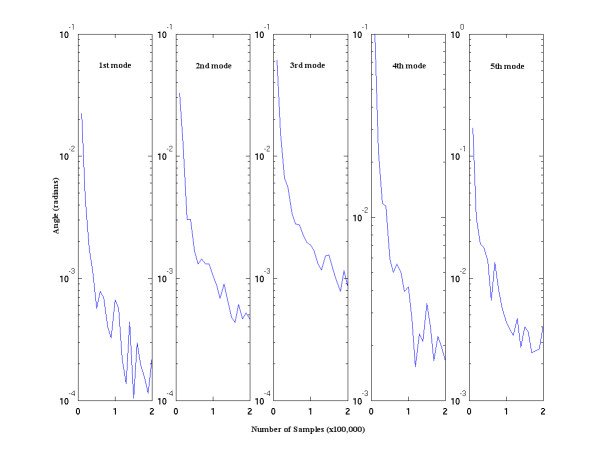
The SVD procedure was applied for increasingly larger sample sizes (up to 200,000 in increments of 10,000) for the metabolic network of the bacterium *Helicobacter pylori *and the first five modes at each sample size were recorded. These plots show the convergence of the angle between the modes for subsequent sample sizes.

While the sampling approach detailed herein used the hRBC for demonstration purposes, the intended application for the method is genome-scale metabolic networks. A requirement for application to such networks is the demonstration of sufficient sampling of their much higher-dimensional steady-state solution spaces. In this section we demonstrate that sufficient sampling is feasible on the genome scale, in the same manner as was done for the hRBC in the Results section above. We use the bacterium *Helicobacter pylori *metabolic reconstruction [28], which is composed of 341 metabolic genes catalyzing 554 reactions amongst 485 metabolites. As can be seen in Figure 8, satisfactory convergence is obtained for the first five modes after 150,000 samples.

## Discussion

In previous work [24] it was demonstrated that SVD of the extreme pathway matrix for the hRBC metabolic network revealed the essential dimensionality of the regulatory problem. Furthermore, it was shown that the dominant features of the eigenpathways corresponding to the five largest singular values corresponded to control points in the hRBC metabolic network. These results are significant because they demonstrate a potentially powerful method for developing network-based understanding of regulation in genome-scale metabolic networks. Unfortunately, the method relies on the computation of the extreme pathways for these networks – a computational exercise that is currently infeasible. In an effort to circumvent this obstacle, we investigated the utility of randomly sampling the steady-state solution space of the hRBC metabolic network, and through SVD of the matrix of sampled solution points arrived at comparable results to those obtained via SVD of **P**. We present three main results. First, we found that the sampling procedure did lead to the same estimates of the dimensionality of the regulatory problem for the metabolic network studied. Secondly, we demonstrated that the resulting PRMs reconstruct physiological flux distributions as efficiently as did the eigenpathways. Thirdly, we showed that the flux adjustments represented by the PRMs were similar, and similarly interpretable, as those represented by the eigenpathways. In particular, we found that the ranking of reactions by their regulatory "importance" in the two approaches was highly correlated with high statistical significance.

It is important to note that there is no *a priori *reason for believing that SVD of **P **would be superior to SVD of **W**. Thus, while the focus of this work was to explain the results of SVD of **W **in the context of what had already been shown for SVD of **P**, it is not necessary that an effort be made to force SVD of **W **to reproduce SVD of **P**. Instead, what was shown herein is that the approaches can be thought of as attacking the same problem and that SVD of **W **provides a way forward into genome-scale matrices to the line of inquiry that was begun with SVD of **P**, since sampling of genome-scale metabolic states has been accomplished at the genome-scale for *E. coli *and *H. pylori *[29,30] – whereas the computation of **P **for these networks has proven infeasible. The results of the two methods will not generally be identical. In the case of SVD of **P **not only the shape of the space, but, more precisely, the distribution of the pathways along the edges of the space is what is being characterized. Because the samples are generated uniformly in the solution space, it is the shape of the solution space alone that is being characterized in this approach. Thus, the interpretation of SVD of **W **is more straightforward than is the interpretation of SVD of **P**.

We used physiological flux distributions computed from a kinetic model and compared how well PRMs and eigenpathways reconstructed them. As can be seen in Figure 7, the PRMs performed just as well, if not better, than the eigenpathways. Indeed, in three instances, the PRMs arrived at essentially complete reconstructions utilizing fewer PRMs. Based on these results, we estimate the regulatory problem for the hRBC to be roughly five-dimensional – in agreement with the estimate based on the eigenpathways.

We compared the PRM and eigenpathway flux distributions in two ways. Figures 2, 3, 4, 5, 6 utilize a flux map of the hRBC and are graphical in manner. Table 3 offers a more concise comparison in that it quantifies the projections of the PRMs and eigenpathways onto one another. In comparing the first five flux maps for the PRMs and eigenpathway fluxes directly we found the first two PRMs and eigenpathways have similar flux distributions (Figures 2 and 3 and Table 3). In the remaining three cases (Figures 4, 5, 6), though, the flux distributions diverge. It can be seen, especially in Table 3, that eigenpathway 3 is more similar to PRM 4 than PRM3, eigenpathway 4 is most similar to PRM 5, and eigenpathway 5 is almost exactly between PRMs 4 and 5. The reason for the divergence of modes is likely due to non-uniform densities of ExPas in the solution space, which since the SVD is a least squares method would have more variation in certain regions than presumably would a randomly sampled space.

The flux maps in Figures 2, 3, 4, 5, 6 demonstrate additional satisfying correspondence between PRMs and eigenpathways. First, the principal singular mode (first PRM) is a valid flux distribution, adhering to mass balance and thermodynamic constraints just as is the case with the principal eigenpathway. Second, the regulatory correspondence that was achieved with the eigenpathways is also possible with the PRMs. For example, the dominant feature of the second PRM is also the flux split between glycolysis and the pentose phosphate pathway. The third singular mode describes, similarly to the third eigenpathway, the glycolytic pathway with the production of ATP, but instead of proceeding through to pyruvate with the production of NADH, it proceeds through to lactate with the utilization of NADH to produce NAD. The fourth PRM, as can be seen in Figure 5, is closer to eigenpathway 5, and this is due to the dominant feature of pyruvate uptake and subsequent conversion to lactate. Finally, the fifth PRM shares the same dominant characterization as eigenpathway 4, this being the flux split between lower glycolysis and the Rapoport-Luebering shunt.

The PRMs of **W **have a slightly different interpretation than the eigenpathways derived from **P**. Mathematically, the modes of **W **are the orthogonal directions of maximal variance in the steady-state solution space of the stoichiometric matrix. Biochemically, they correspond to the directions of greatest flexibility in achieving a network-wide flux distribution. The unequal variances in these orthogonal directions imply a hierarchy, wherein control of the more dominant modes gives the cell more control of achieving a desired flux distribution.

## Conclusion

From these results we conclude that SVD of a sample of uniformly obtained steady state flux distributions for a metabolic network leads to a similar means of characterizing the regulatory problem as was obtained previously with ExPas. This was a necessary demonstration on a small system, before applying the technique to genome-scale networks. With a number of genome-scale metabolic networks now significantly reconstructed for a number of organisms, the results presented in this work indicate that it should now be possible – given that feasible genome-scale sampling has been demonstrated [29,30] – to realize the dimensionality and mechanisms of metabolic regulation implicit in the topology of these networks.

## Methods

### Human red blood cell model

For this work we utilized a previously-constructed kinetic model [31] and a stoichiometric model [22,24] of the human red blood cell. The stoichiometric model accounts for 39 metabolites and 32 internal metabolic reactions, and also contains 12 primary exchange fluxes and 7 currency exchange fluxes.

### Physiologic steady-state flux distributions

Four steady-state flux distributions were calculated using an *in silico *kinetic model [31], as detailed in earlier work [24], and used to represent physiological states of interest in the solution space. The first distribution was for an unstressed state or normal physiological state, corresponding to no metabolic load. The other three corresponded to the primary metabolic demands placed upon the hRBC based on its physiologic role in the human body. These demands were for ATP (energy load), NADPH (oxidative load), and NADH (met-hemoglobin load). In each case, the load was maximized in the kinetic model to the highest value possible where the model could still be solved. Thus, they represented as high a load as the network could handle. All these states were previously computed and used in a previous study [24]. These maximum values were 0.37 mM/h for ATP load (above a mandatory ~1 mM/hr running of the Na^+^/K^+ ^pump), 2.5 mM/h for the NADPH load, and 1.7 mM/h for the NADH load.

### Physiologic flux constraints

The null space of the stoichiometric matrix of the hRBC metabolic network is a (mathematically) unbounded space. To transform this space into one that is physiologically relevant, we applied, as before, constraints by bounding the minimum and maximum fluxes for each reaction. All irreversible reactions were constrained with a minimum flux of 0 mM/h and a maximum flux of 2000 mM/h unless experimentally-determined maximum flux values were available. Such maximum reaction rates were available for 22 reactions, and have been published previously [22,32]. Additionally, certain fluxes must have a minimum, non-zero value in order to sustain the hRBC. These values were obtained from a previous study on the hRBC [32,33], and since they are few in number are given here: DPGase, 0.3 mM/h; ATP production, 1 mM/h; NADH production, 0.4 mM/h; and NADPH production, 0.05 mM/h.

### Hit-and-Run sampling of the solution space

We use a variant of an artificial centering hit-and-run algorithm [34] to sample random flux distributions in the steady-state solution space of the hRBC. The algorithm is a random walk algorithm entailing three steps. The first step is to generate a set of "warmup" points in the solution space. This generation of warmup points was performed by slightly reducing the maximum flux and increasing the minimum flux for all reactions and then calculating a candidate flux distribution along a reaction coordinate using linear programming. Ten thousand such warmup points were created. The second step of the algorithm is to select an initial point from one of the "warm up" points generated. The warm up points were stored in a matrix **W **and were used to calculate an approximate center ***s ***of the solution space. The third step calculates the sample points. Beginning with an initial, randomly selected point ***x***_*m *_from **W**, a direction vector is constructed by choosing a randomly selected point *y *from **W **and moving along the direction vector **d **= **y**-**s **from ***x***_*m *_resulting in a new point ***x***_*m*+1_. After each such move ***x***_*m*+1 _was randomly substituted into **W **and the approximate center recalculated. For the results presented herein, we saved a sample point every 1000 moves in the space. It should be noted that the red blood cell metabolic network does not have any non-trivial biochemical loops (type III ExPas) [35], and thus the samples generated do not violate "loop law" thermodynamic constraints [36,37] that would otherwise need to be explicitly addressed in sampling [30]. Note that in previous work [22], it was stated that the hRBC contains 16 Type III ExPas. The computation of ExPas in that work entailed the decoupling of reversible reactions into forward and backward reactions, and these resulting trivial loops were the 16 Type III pathways. In this work, reversible reactions were not decoupled, and so trivial Type III pathways were not an issue. One issue not encountered in this work which will is important for sampling genome-scale networks is the issue of non-trivial Type III ExPas (more than 2 reactions). Since Type III ExPas correspond to thermodynamically infeasible flux distributions, the generation of samples from genome-scale systems will need to be cognizant of this issue. This issue has been addressed elsewhere and will not be an impediment to sampling genome-scale metabolic networks [30].

### Extreme pathways

The extreme pathways were calculated from the stoichiometric matrix of the hRBC using a previously published algorithm [2]. V_max _values for the reactions in the hRBC have been previously published [22,32]. Each extreme pathway was scaled by the most limiting V_max _amongst all reactions that it contained. All of the extreme pathways had at least one reaction with an experimentally defined V_max_.

### Angle between vectors

Angles between modes were computed using standard linear algebra. For two vectors **u **and **v**, the angle was computed as Θ = cos^-1 ^(**u**·**v**/||**u**|| ||**v**||).

### Regulatory importance of reactions

The result of applying the SVD to either the matrix of ExPas or the matrix of uniformly sampled flux distributions is a set of eigenvectors. Each reaction has a corresponding loading vector, which is the vector of the reaction's contribution to each of the eigenvectors. Specifically, if v_ij _is the contribution of reaction *j *to eigenvector *i*, and there are *m *eigenvectors, then the loading vector for reaction *j *is **l**_*j *_= (v_1*j*_, v_2*j*_, ... , v_*mj*_). The score for reaction *j *is then given by the *l*^2^-norm of the loading vector: | **l**_*j *_| = (v_1*j*_^2 ^+ v_2*j*_^2 ^+ ... + v_*mj*_^2^)^1/2^.

## Abbreviations

SVD, singular value decomposition; ExPa, extreme pathway; PRM, principal regulatory mode

## Authors' contributions

CB and NP performed the sampling and interpretation of the results. CB drafted the manuscript and NP and BP provided critical edits.

## Figures and Tables

**Table 1:** Metabolite abbreviations used in Figures 2–6.

**Abbr.**	**Metabolite**	**Abbr.**	**Metabolite**
13DPG	1,3-diphosphoglycerate	GLU	glucose
23DPG	2,3-diphosphoglycerate	HX	hypoxanthine
2PG	2-phosphoglycerate	IMP	inosine monophosphate
3PG	3-phosphoglycerate	INO	inosine
6PGC	phosphogluconate	LAC	lactate
6PGL	phosphogluco-lactone	PEP	phosphenolpyruvate
ADE	Adenine	Pi	inorganic phosphate
ADO	Adenosine	PRPP	5-phosphoribosyl-1-pyrophosphate
DHAP	dihydroxyacetone phosphate	PYR	pyruvate
E4P	erythrose-4-phosphate	R1P	ribose-1-phosphate
F6P	fructose-6-phosphate	R5P	ribose-5-phosphate
FDP	fructose-1,6-bisphosphate	RL5P	ribulose-5-phosphate
G6P	glucose-6-phosphate	S7P	sedoheptulose-7-phosphate
GA3P	glyceraldehyde-3-phosphate	X5P	xylulose-5-phosphate

**Table 2:** Reaction abbreviations used in Figures 2–6. The corresponding reaction stoichiometries are also included.

**Abbr.**	**Enzyme Name**	**Metabolic Reaction**
ADA	Adenosine deaminase	ADO + H2O → INO + NH3
AdPRT	Adenine phosphoribosyl transferase	PRPP + ADE + H2O → AMP + 2 Pi
AK	Adenosine kinase	ADO + ATP → ADP + AMP
ALD	Aldolase	FDP ↔ GA3P + DHAP
AMPase	Adenosine monophosphate phosphohydrolase	AMP + H2O → ADO + Pi + H
AMPDA	Adenosine monophosphate deaminase	AMP + H2O → IMP + NH3
ApK	Adenylate Kinase	2 ADP ↔ ATP + AMP
DPGase	Diphosphoglycerate phosphatase	23DPG + H2O → 3PG + Pi
DPGM	Diphosphoglyceromutase	13DPG → 23DPG + H
EN	Enolase	2PG ↔ PEP + H2O
G6PDH	Glucose-6-phosphate dehydrogenase	G6P + NADP → 6PGL + NADPH + H
GAPDH	Glyceraldehyde phosphate dehydrogenase	GA3P + NAD + Pi ↔ 13DPG + NADH + H
HGPRT	Hypoxanthine guanine phosphoryl transferase	PRPP + HX + H2O → IMP + 2 Pi
HK	Hexokinase	GLU + ATP → G6P + ADP + H
IMPase	Inosine monophosphatase	IMP + H2O → INO + Pi + H
LD	Lactate dehydrogenase	PYR + NADH + H ↔ LAC + NAD
PDGH	6-phosphoglycononate dehydrogenase	6PGC + NADP → RL5P + NADPH + CO2
PFK	Phosphofructokinase	F6P + ATP → FDP + ADP + H
PGI	Phosphoglucoisomerase	G6P ↔ F6P
PGK	Phosphoglycerate kinase	13DPG + ADP ↔ 3PG + ATP
PGL	6-phosphoglyconolactonase	6PGL + H2O ↔ 6PGC + H
PGM	Phosphoglyceromutase	3PG ↔ 2PG
PK	Pyruvate kinase	PEP + ADP + H → PYR + ATP
PNPase	Purine nucleoside phosphorylase	INO + Pi ↔ HX + R1P
PRM	Phosphoribomutase	R1P ↔ R5P
PRPPsyn	Phosphoribosyl pyrophosphate synthetase	R5P + ATP → PRPP + AMP
RPI	Ribose-5-phosphate isomerase	RL5P ↔ R5P
TA	Transaldolase	GA3P + S7P ↔ E4P + F6P
TK1	Transketolase	X5P + R5P ↔ S7P + E4P
TK2	Transketolase	X5P + E4P ↔ F6P + GA3P
TPI	Triose phosphate isomerase	DHAP ↔ GA3P
XPI	Xylulose-5-phosphate epimerase	RL5P ↔ X5P

**Table 3:** The fractional projection of eigenpathways and PRMs onto one another.

from\onto	eigpath1	eigpath2	eigpath3	eigpath4	eigpath5	PRM1	PRM2	PRM3	PRM4	PRM5
eigpath1	0	0.021	0.021	0.126	0.384	0.950	0.107	0.077	0.247	0.054
eigpath2	0.022	0	0.039	0.187	0.300	0.028	0.983	0.016	0.195	0.168
eigpath3	0.025	0.045	0	0.011	0.252	0.212	0.189	0.671	0.899	0.231
eigpath4	0.114	0.165	0.009	0	0.108	0.049	0.029	0.280	0.378	0.763
eigpath5	0.387	0.295	0.214	0.120	0	0.282	0.284	0.430	0.584	0.560
PRM1	0.957	0.027	0.180	0.055	0.282	0	0	0	0	0
PRM2	0.108	0.966	0.160	0.033	0.284	0	0	0	0	0
PRM3	0.078	0.016	0.569	0.311	0.430	0	0	0	0	0
PRM4	0.248	0.192	0.761	0.421	0.584	0	0	0	0	0
PRM5	0.055	0.165	0.195	0.848	0.560	0	0	0	0	0
